# Sensing and Adaptation to Low pH Mediated by Inducible Amino Acid Decarboxylases in *Salmonella*

**DOI:** 10.1371/journal.pone.0022397

**Published:** 2011-07-25

**Authors:** Julie P. M. Viala, Stéphane Méresse, Bérengère Pocachard, Aude-Agnès Guilhon, Laurent Aussel, Frédéric Barras

**Affiliations:** 1 Laboratoire de Chimie Bactérienne, Institut de Microbiologie de la Méditerranée, CNRS (UPR-CNRS 9043), Marseille, France; 2 Centre d'Immunologie de Marseille-Luminy, UMR-CNRS 6102–INSERM U631-Univ. Méditerranée, Marseille, France; 3 Aix-Marseille Université, Faculté des Sciences, Marseille, France; Indian Institute of Science, India

## Abstract

During the course of infection, *Salmonella enterica* serovar Typhimurium must successively survive the harsh acid stress of the stomach and multiply into a mild acidic compartment within macrophages. Inducible amino acid decarboxylases are known to promote adaptation to acidic environments. Three low pH inducible amino acid decarboxylases were annotated in the genome of *S*. Typhimurium, AdiA, CadA and SpeF, which are specific for arginine, lysine and ornithine, respectively. In this study, we characterized and compared the contributions of those enzymes in response to acidic challenges. Individual mutants as well as a strain deleted for the three genes were tested for their ability (i) to survive an extreme acid shock, (ii) to grow at mild acidic pH and (iii) to infect the mouse animal model. We showed that the lysine decarboxylase CadA had the broadest range of activity since it both had the capacity to promote survival at pH 2.3 and growth at pH 4.5. The arginine decarboxylase AdiA was the most performant in protecting *S*. Typhimurium from a shock at pH 2.3 and the ornithine decarboxylase SpeF conferred the best growth advantage under anaerobiosis conditions at pH 4.5. We developed a GFP-based gene reporter to monitor the pH of the environment as perceived by *S*. Typhimurium. Results showed that activities of the lysine and ornithine decarboxylases at mild acidic pH did modify the local surrounding of *S*. Typhimurium both in culture medium and in macrophages. Finally, we tested the contribution of decarboxylases to virulence and found that these enzymes were dispensable for *S*. Typhimurium virulence during systemic infection. In the light of this result, we examined the genomes of *Salmonella spp.* normally responsible of systemic infection and observed that the genes encoding these enzymes were not well conserved, supporting the idea that these enzymes may be not required during systemic infection.

## Introduction

*Salmonella enterica* serovar Typhimurium is a food-borne, facultative intracellular pathogen, which triggers a typhoid-like systemic infection in the mouse model. Upon ingestion, *S.* Typhimurium must first resist the very acidic pH (∼pH 2) in the stomach of the infected host [1]. Then, bacteria cross the intestinal barrier and invade deeper organs such as the spleen and the liver where *S.* Typhimurium replicates in cells of the monocytic lineage [2]. Inside host cells, *S.* Typhimurium proliferates into a compartment called the *Salmonella*-containing vacuole (SCV). The pH value of the SCV has been estimated to be below 5 in macrophages [3], [4], [5]. This acidic pH is a signal that triggers the activation of crucial virulence systems such as the PhoPQ two-component system [6] and the type three secretion system encoded by the *Salmonella* pathogenicity island II [7]. Both are necessary for survival and proliferation inside host cells [8], [9], [10]. Acidification of the SCV is therefore necessary for intracellular proliferation [5], [7]. Thus, *Salmonella* growth exhibits two pH values optima: ∼7 as a free bacteria growing in laboratory standard conditions, and ∼4.5-5 as an intracellular pathogen growing into macrophages [5].

In *S*. Typhimurium, global transcriptional regulators, such as the two-component regulators PhoP and OmpR, the sigma factor RpoS and the iron regulator Fur, contribute to the protection against acid stress. However, members of those regulons that are specifically involved in acid protection are not yet clearly identified. On the other hand, systems involving an antiporter and an associated amino acid decarboxylase also protect *S.* Typhimurium from an acid shock [11], [12]. These systems are partly induced by low pH [13], [14], [15] and the decarboxylases are therefore named inducible or biodegradative amino acid decarboxylases to distinguish them from the biosynthetic ones involved in polyamine synthesis at neutral pH. Inducible amino acid decarboxylases are pyridoxal phosphate-containing enzymes that replace the α-carboxyl groups of their cognate amino acid substrates with a proton consumed from the cytoplasm:
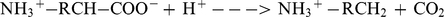



Subsequently, the reaction product is secreted *via* the corresponding antiporters and exchanged for a new substrate. Consumption of internal protons and release of a reaction product, which is a di- or triamine, provide local buffering of the extracellular environment. *S.* Typhimurium possesses three inducible amino acid decarboxylases: the arginine (AdiA), lysine (CadA) and ornithine (SpeF) decarboxylases. Decarboxylation of arginine, lysine and ornithine leads to the production of agmatine, cadaverine and putrescine, respectively [1]. Both the arginine and lysine decarboxylase systems have been involved in survival at extremely acidic pH [13], [14], [16]. However their contribution during growth at moderate acidic pH has not been reported and no study has yet been published on the ornithine decarboxylase. Expression of the arginine-dependent system is induced by low-pH and anoxic conditions [13], and the lysine-dependent system is expressed in low pH medium containing lysine [14]. Expression of members of the arginine- and lysine-dependent systems has been specifically detected in infected cultured cells or in animal host [17], [18], [19]. Hence, inducible amino acid decarboxylases appear to be active during infection and a reasonable hypothesis would be that they protect *S*. Typhimurium from the various acid challenges.

In this study, we made a comparative analysis of the contribution of the three inducible amino acid decarboxylases of *S.* Typhimurium in response to acidic stresses. Each individual mutants and a strain deleted for the three genes *adiA, cadA* and *speF* were monitored for survival at extreme acidic pH and growth at moderate acidic pH. We took advantages of the bacterial pathogen *S.* Typhimurium, for which exist cellular and animal models, to examine if the decarboxylases contributed to virulence.

We showed that *S.* Typhimurium inducible amino acid decarboxylases promoted survival at pH 2.3 with the following efficiency, AdiA > CadA > SpeF. We also showed that CadA and SpeF promoted growth at pH 4.5. Developing a reporter system to follow the environmental pH as perceived by the bacterium, we observed that activities of the decarboxylases influenced the environmental pH both in culture and in the SCV. However, our results indicated that the absence of the decarboxylases was not detrimental to the bacterium during systemic infection in the mouse model.

## Methods

### Bacterial strains, growth and stress conditions

The bacterial strain used in this study was *Salmonella enterica* subspecies *enterica* serovar Thyphimurium 12023 (laboratory stock). Mutants derived from the parental strain *S.* Typhimurium 12023 were: Δ*adiA*::Kn^R^ (strain n° 84), Δ*cadA*::Cm^R^ (strain n° 78), Δ*speF*::Kn^R^ (strain n° 193), Δ*adiA*::Kn^R^Δ*speF* (strain n° 221), Δ*adiA*Δ*cadA*Δ*speF* Kn^S^ (strain n° 197) and Δ*adiA*::Kn^R^Δ*cadA*Δ*speF* (strain n° 199). The strain Δ*adiA*Δ*cadA*Δ*speF* n° 197 was used in all experiments except the competitive index in mice for which we needed an antibiotic resistant strain and for which we therefore used the strain Δ*adiA*Δ*cadA*Δ*speF* Kn^R^ n° 199.

Media used to grow bacteria were Luria-Bertani (LB) (Sigma-Aldrich) or Luria-Bertani Glucose (LBG) containing 0.4% Glucose. Ampicillin (50 µg/ml) and kanamycin (25 µg/ml) were added when necessary.

For growth at pH 4.5, overnight cultures grown in LBG in aerobic or anoxic conditions were washed and suspended to an OD_600_ = 0.03 in minimal medium (M9) supplemented with MgSO_4_ (1 mM), CaCl_2_ (200 µM), thiamine (10^-4^%), 0.1% casamino acids, 0.2% glucose and adjusted to the desired pH with hydrochloric acid (HCl). For the one hour challenge at pH 2.3, overnight cultures grown in LBG pH 5 in anoxic conditions were washed and diluted 1/1000 in M9 medium with the following modifications: 0.4% glucose, no casamino acids and pH 2.3. Amino acids L-lysine monohydrochloride, L-ornithine monohydrochloride and L-arginine monohydrochloride (Sigma-Aldrich) were added in the medium at 5 mM for growth at pH 4.5 and 20 mM for challenge at pH 2.3, then the pH was controlled and adjusted. In aerobic conditions, bacteria were grown in a flask 5 to 10 times the culture volume with agitation at 150 rpm. In anoxic conditions, bacteria were grown in a 10 ml culture plastic tube completely filled, closed with a hermetic cap and without aeration. Tubes were never opened throughout the experiment as they fitted into the spectrophotometer.

Stress applied to check the specificity of response to acidic pH by the *asr* promoter were 10 µM instead of 1 mM magnesium, 50 µM dipyridil (Sigma-Aldrich) for the iron starvation, no casamino acids for amino acid starvation, 50 µM H_2_O_2_ (Sigma-Aldrich) for oxidative stress and 0.75 µg/ml polymyxin B (Fluka) for antimicrobial peptides.

### DNA manipulations

Gene deletions were carried out using Lambda-Red-mediated recombination [20]. Briefly, the coding sequences from ATG to the Stop for *cadA* and *adiA* or to 90 bp before the Stop for *speF* were deleted by homologous recombination using PCR products made with primers 298-299, 302-303 and 306-392, respectively. Those primers had a 40 bases 5′-end homologous to the gene of interest, necessary for recombination, and a 20 bases 3′-end homologous to plasmids pKD3 and pKD4, necessary for amplification of the antibiotic resistance gene. PCR products were used to transform *S.* Typhimurium 12023 harboring the plasmid pKD46 from which the λ red recombinase was expressed. Mutations were moved to wild-type or mutant background by P22 transduction. When necessary, antibiotic resistance cassettes were removed by using the temperature sensitive plasmid pCP20 carrying the FLP recombinase [20].

The promoter-*gfp* fusion was made on plasmid pFPV25 [21] by inserting an *Eco*R I- *Xba* I fragment including the promoter of the *asr* gene (STM1485) [22]. The construction was called pP*asr::gfp* (plasmid n°82).

The vector used for complementation was pACYC177 [23], in which a PCR product corresponding to *cadA* under the control of its own promoter was introduced at the *Sma* I and *Hin*d III sites. As *cadA* is the second gene of the *cadBA* operon, *cadA* and the cognate promoter was amplified from a Δ*cadB* strain, from which the antibiotic resistance cassette had been removed (strain n° 190, JV, unpublished). Primers used for the amplification were primers 526 and 301. The construction was called p*cadA* (plasmid n°118)

Primers used for chromosomal deletions and plasmid constructions are listed in Table 1.

**Table 1:** List of primers.

Name	Sequence 5′-3′	Purpose
298	TAGCGTAGCGGGAGGGGCCCACTTTACCAGGAACAAGACTtgtgtaggctggagctgcttc	*cadA* deletion
299	CGTGAAAAAAGGGAAGTGGCAAGCCACTTCCCTTTGGTACcatatgaatatcctccttag	
302	GCAAGGCGTAAATTGCACGGCCTCCACAACCGGGTAAAAGtgtgtaggctggagctgcttc	*adiA* deletion
303	CAGGGAATACATGCCATCCTCAAAAAAAAGACTCTTTGTGAcatatgaatatcctccttag	
306	AATTGAGGGCCTGCTATTACCTGAAATAAAGAGATGAAAAtgtgtaggctggagctgcttc	*speF* deletion
392	GACGTAGCACCAGACCTGCTTACGGCCGTCGTGTTCTTCGcatatgaatatcctccttag	
273	gaagaattcAAAGAAATAATCCGGCGATAG	pP*asr::gfp* construction
274	tcttctagaGATTTGGTTTTCATTCAACCC	
526	aaaaagcttATTTAACGCTGAACCATGAC	p*cadA* construction
301	CGCCACGATGTAAAAAATCG	

Capital letters correspond to *S.* Typhimurium chromosomal sequence. Restriction endonuclease sites are underlined.

### Bacterial infection of macrophages

RAW 264.7 macrophages were cultivated in DMEMs made of DMEM (Lonza) supplemented with glutamine 2 mM, non-essential amino acids 0.1 mM (Gibco) and 10% fetal calf serum (HyClone). For infection, cells were seeded in 6-well tissue culture plates at a density of 0.5×10^6^ cells per well. Bacteria were cultured overnight in LB, washed once and opsonized in DMEMs 10% normal mouse serum (Perbio) for 30 minutes on ice. When indicated, Bafilomycin A1 (Sigma-Aldrich) was added at 100 nM, 15 min before infection and throughout the experiment. Bacteria were added to the monolayers at a M.O.I. of 50:1, centrifuged at 400 *g* for 5 min, and incubated for 30 min at 37°C in 5% CO_2_. Then, macrophages were washed three times with Dulbecco's phosphate buffered saline (DPBS) (Lonza), and incubated with DMEMs containing 100 µg/ml gentamicin (Sigma-Aldrich) for 90 minutes, after which the gentamicin concentration was decreased to 10 µg/ml for the remainder of the experiment. At different time post-infection, macrophages were washed twice with DPBS, lysed with a DPBS 0.1% Triton X-100 solution and lysates containing intracellular bacteria were transferred to 3.2% paraformaldehyde (Euromedex). Bacterial fluorescence was subsequently analyzed by flow cytometry.

To monitor impact of the intracellular activity of the decarboxylases on the surrounding pH values, when indicated cells were incubated in DMEMs containing 20 mM L-lysine and L-ornithine 3 h before infection and throughout the experiment. Bacteria were cultured overnight and sub-cultured 1/100 for 5 hours at 37°C in LBG pH 7 in anoxic conditions, washed once and opsonized in DMEMs 10% normal mouse serum (Perbio) for 30 minutes on ice. The rest of the experiment was performed as described above.

### Fluorescence analysis with microplate reader or flow cytometer

Fluorescence from bacteria grown in M9 medium was analyzed with a microplate reader Infinite 200 (TECAN). Bacteria were grown in 96-well plate at 37°C with of without aeration. Fluorescence detected with a 482 and 515 nm excitation and emission wavelengths, respectively, was divided by the absorbance at 600 nm to make it proportional to bacterial cell concentration.

Fluorescence from bacteria isolated from infected macrophages was analyzed by flow cytometry. Lysates from infected macrophages were centrifuged 5 min at 100 g to remove cell debris. Supernatants were saved and centrifuged 5 min at 13 000 rpm. Pellets were suspended in PBS 10 mM NH_4_Cl. Mouse monoclonal antibody 1E6 (Interchim) directed against lipopolysaccharides of *S.* Typhimurium, followed 20 min later by a phycoerythrin conjugated secondary donkey anti-mouse antibody (Jackson ImmunoResearch) were added at a 1/2000 dilution. Samples were then analyzed with a FACScalibur flow cytometer (Becton Dickinson) equipped with a blue argon laser (488 nm). For analysis of bacterial cells, samples were gated for *S*. Typhimurium particles based on the phycoerythrin fluorescence. Data were processed with a FlowJo software on 2000 to 10000 events finally identified as *S.* Typhimurium particles.

### Competitive index in mouse

Bacteria were grown 5 hours in LBG pH 5 and anoxic conditions. Eight- to 10 week-old C57/B6 mice were inoculated intragastrically with equal amounts of wild-type and mutant strains for a total of 2.5×10^4^ bacteria per mouse diluted in M9 pH 5. The amino acid substrate(s) susceptible to be used by the WT but not the mutant strain was delivered at the time of inoculation. Amino acids were at a concentration of 20 mM each. The spleens were harvested 5 days after inoculation and homogenized. Bacteria were recovered after plating a dilution series onto LB agar. Between 200 and 350 clones, from the input (initial inoculum) and the output (bacteria recovered from the mouse after infection), were patched on LB agar with the appropriate antibiotic to estimate the number of wild-type antibiotic sensitive and mutant antibiotic resistant bacteria. Competitive indexes (CI) were determined for each mouse, three to four mice were inoculated and the experiment sometimes repeated twice for each competition test. The CI is defined as the ratio between the mutant and wild-type strains within the output divided by their ratios within the input. Unpaired *t* test analysis was performed to compare two CIs, and a one-sample *t* test comparing the log of the CI to 0 was used to determine whether the CI was significantly different from 1. All statistical analyses were performed using Prism (GraphPad, San Diego, CA). The two-tailed *P* value was calculated and a *P* value <0.05 indicated a CI significantly different from 1.

### Ethics statement

Animal experimentation was conducted in strict accordance with good animal practice as defined by the French animal welfare bodies (Law 87–848 dated 19 October 1987 modified by Decree 2001-464 and Decree 2001-131 relative to European Convention, EEC Directive 86/609). All animal work was approved by the Direction Départementale des Services Vétérinaires des Bouches du Rhône (authorization number 13.118 to S.M.).

## Results

### Activities of the decarboxylases improve survival at pH 2.3

Capacity of inducible amino acid decarboxylases to promote survival at acidic pH was investigated. For this, four strains were made. Individual mutants of *adiA*, *cadA* and *speF* encoding arginine, lysine and ornithine decarboxylases, respectively, were constructed. A strain referred to as Δ*adiA*Δ*cadA*Δ*speF*, in which all the three genes were deleted, was also made (see Materials and Methods).

Bacteria were cultivated at pH 5 and then challenged for 1 hour at pH 2.3. Cultures and challenges were done in anoxic conditions, which are known to increase expression of inducible amino acid decarboxylases [13], [24]. Substrate amino acids, arginine, lysine and ornithine, were made available in the medium during the challenge and the non-substrate amino acid glutamine was used as a negative control. Survival was assessed by plating bacteria, and by counting CFU before and after challenge at pH 2.3. Survival efficiency of the WT strain was greatly improved by the addition of arginine, significantly improved by the addition of lysine, modestly improved by the addition of ornithine and not improved at all by the addition of the non-substrate amino acid glutamine (Table 2). Examination of individual mutants showed that they were altered in the use of their cognate substrate to promote survival. Surprisingly, Δ*cadA* also showed less survival in presence of ornithine. While this might point to a cross-talk between the two systems, further investigation would be required to understand this observation. The Δ*adiA*Δ*cadA*Δ*speF* strain did not show any survival at pH 2.3 in any of tested conditions. The survival mediated by decarboxylases at pH 2.3 was strictly dependent on anoxic conditions during growth and challenge (data not shown). These results showed that the contribution to survival at extreme acidic pH was mainly carried by arginine and lysine decarboxylases.

**Table 2:** Survival after a stress of one hour at pH 2.3.

Strain	Available substrate				
	None	Arginine	Lysine	Ornithine	Glutamine
**WT**	0.6±0.5	103±23	61±16	20±17	0.8±0.8
**Δ** ***adiA***	0.5±0.6	0.6±0.6	58±20	10±6	NT
**Δ** ***cadA***	0.2±0.3	120±26	0.5±0.7	1±1	NT
**Δ** ***speF***	0.9±0.5	81±35	48±14	3±2	NT
**Δ** ***adiA*** **Δ** ***cadA*** **Δ** ***speF***	<0.05	<0.05	<0.05	<0.05	<0.05

Values in the table are percentage survival ± standard deviation, which results from three independent experiments. Viability was assessed by CFU count on LB plates. The percentage survival represents the ratio between the CFU counted after and before stress. NT means not tested. Bacteria grown overnight in LBG pH 5 were diluted 1/1000 in M9 acidified to pH 2.3 and challenged for one hour. Cultures and stresses were done in anoxic conditions. When indicated 20 mM L-Arg, L-Lys, L-Orn or L-Gln were added to the medium during the challenge.

### Activities of the decarboxylases improve growth at pH 4.5

**Figure 1:**
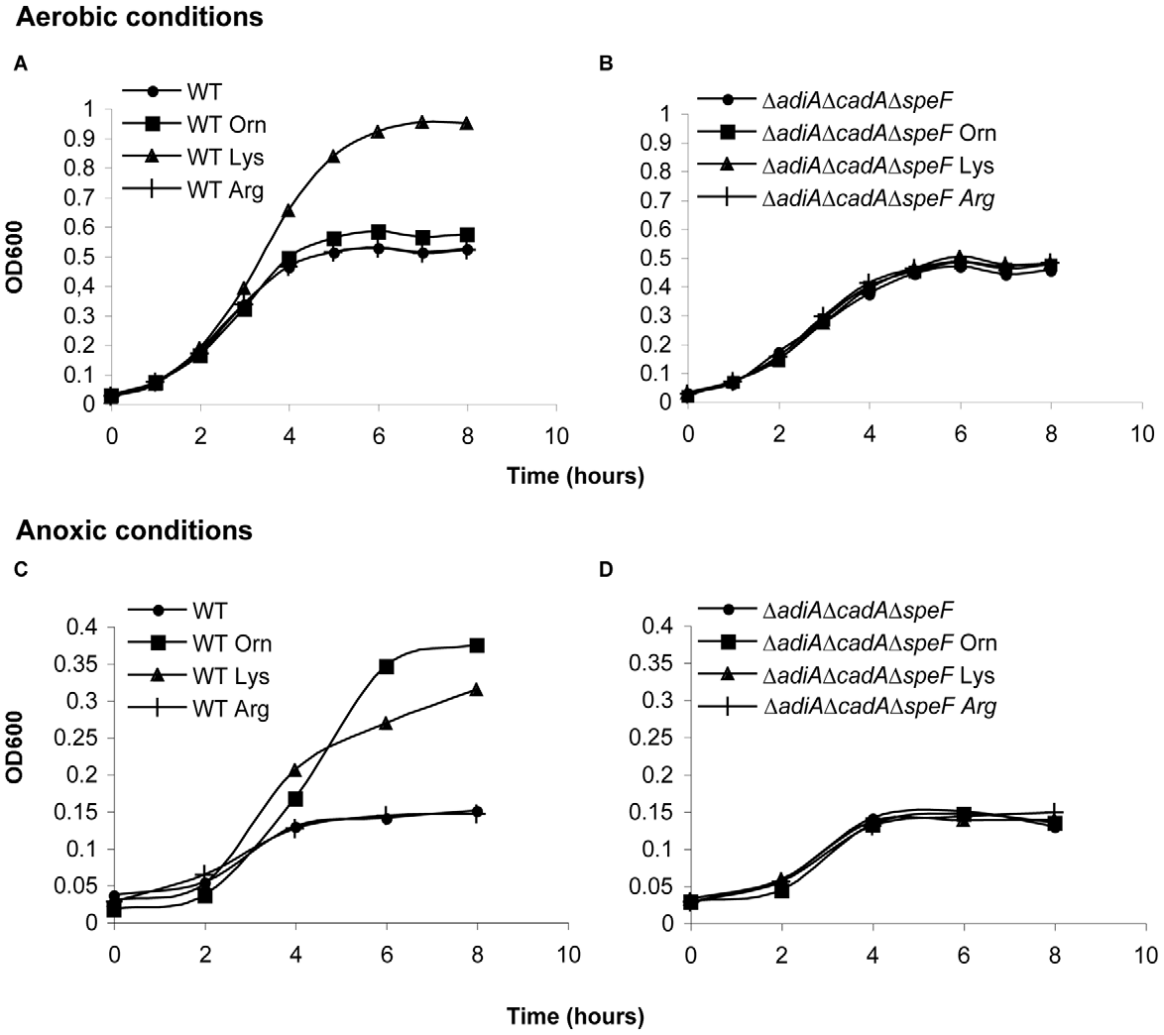
Growth of WT and Δ*adiA*Δ*cadA*Δ*speF* at moderate acidic pH. Bacteria grown overnight in LBG pH 7 were washed and diluted to OD_600_ = 0.03 in M9 medium complemented with 0.1% casamino acids, 0.2% glucose and adjusted to pH 4.5 with HCl. When indicated 5 mM L-ornithine, L-lysine or L-arginine were added to the medium. Cultures were performed in aerobic (A-B) or anoxic (C-D) conditions and monitored by following optical density at 600 nm. Typical growth curves representative of several experiments are shown. WT is the wild-type strain; Δ*adiA*Δ*cadA*Δ*speF* is the strain deleted for the three genes *adiA, cadA,* and s*peF* (strain n°197).

**Figure 2:**
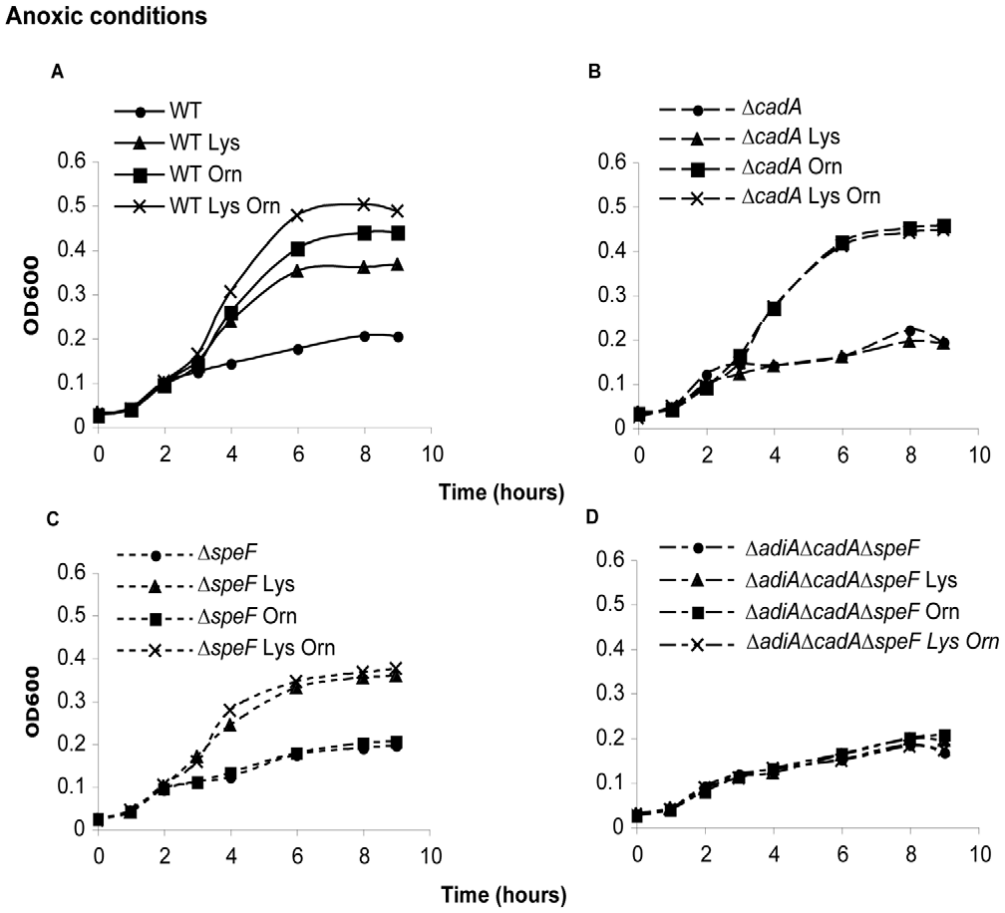
Growth of the Δ*cadA* and Δ*speF* mutants in anoxic conditions at moderate acidic pH. Bacteria grown overnight in LBG pH 7 were washed and diluted to OD_600_ = 0.03 in M9 medium complemented with 0.1% casamino acids, 0.2% glucose and adjusted to pH 4.5 with HCl. When indicated 5 mM L-ornithine and/or 5 mM L-lysine were added to the medium. Cultures were performed in anoxic conditions and monitored by following optical density at 600 nm.

We asked whether activity of inducible amino acid decarboxylases could help bacteria to grow at moderate acidic pH. Bacteria were cultivated and grown under different aeration conditions at pH 4.5. Under aerobic conditions, only the addition of lysine in the medium improved growth of the WT strain (Figure 1A). But under anoxic conditions, both addition of lysine and ornithine improved growth of the WT strain (Figure 1C). Arginine added to the medium never conferred any advantages at this pH (Figure 1A-C). The Δ*adiA*Δ*cadA*Δ*speF* strain did not show any growth improvement in response to the addition of lysine or ornithine in the medium (Figure 1B-D). We then studied how individual mutants Δ*cadA* and Δ*speF* responded to the presence of lysine and ornithine in the medium at pH 4.5. The Δ*cadA* mutant was only impaired in its ability to use its own substrate lysine while the Δ*speF* mutant was only altered in its ability to use ornithine. The growth benefit conferred by decarboxylases was specific of acidic conditions as addition of lysine or ornithine had no effect at pH 7 (data not shown). Of note, decarboxylases were not strictly required to grow at pH 4.5 since the WT and mutant strains grew similarly in the absence of substrates of the decarboxylases (Figure 1 and 2). These results showed that ornithine and lysine decarboxylase activities improved growth at moderate acidic pH, provided that their substrates were available. This growth improvement was observed exclusively in anoxic conditions for the ornithine decarboxylase and independently of O_2_ conditions for the lysine decarboxylase.

### Complementation by *cadA* restores growth improvement and survival

In our experiments, the lysine decarboxylase CadA conferred advantages in a broader range of conditions than the other two decarboxylases AdiA and SpeF. Therefore, we tested if the sole expression of *cadA* was sufficient to confer survival at extreme acidic pH and growth improvement at moderate acidic pH. For that purpose, we took advantage of the Δ*adiA*Δ*cadA*Δ*speF* strain, which we transformed with a plasmid containing the *cadA* gene.

**Figure 3:**
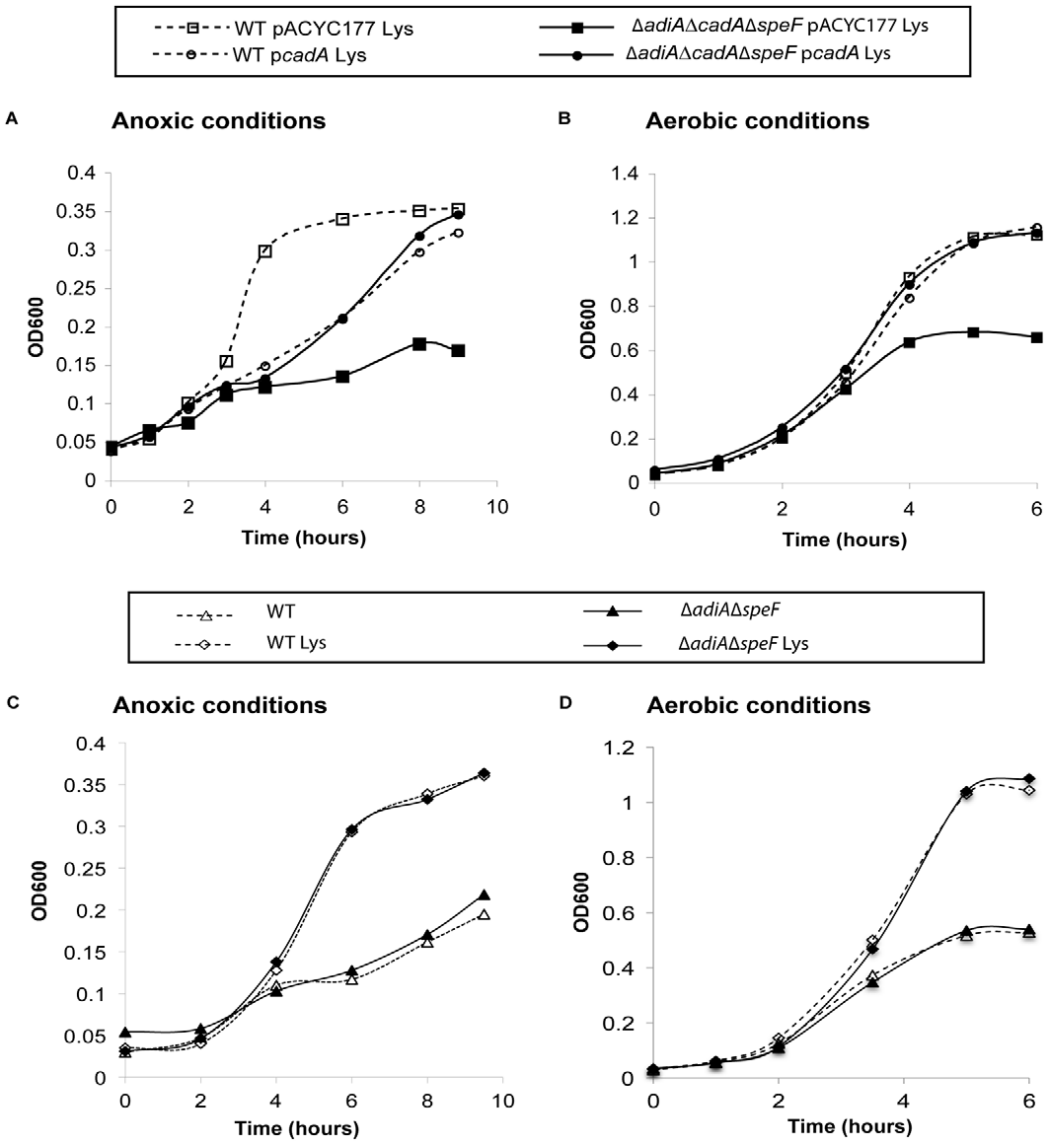
Complementation by p*cadA* and analysis of Δ*adiA*Δ*speF* during growth at moderate acidic pH. Bacteria grown overnight in LBG pH 7 were washed and diluted to OD_600_ = 0.03 in M9 medium complemented with 5 mM L-lysine, 0.1% casamino acids, 0.2% glucose and adjusted to pH 4.5 with HCl. pACYC177 is a low-copy cloning vector and p*cadA* is pACYC177 in which the *cadA* gene has been introduced under control of its natural promoter. Cultures were either performed in anoxic (A) or aerobic (B) conditions and monitored by following optical density at 600 nm. Panels C and D show growth of the WT and Δ*adiA*Δ*speF* strains in the same M9 medium complemented or not with 5 mM lysine, at pH 4.5 in anoxic (C) and aerobic (D) conditions.

The *cadA* gene was cloned under the control of its own promoter into the low copy number vector pACYC177 to create p*cadA*. The empty vector pACYC177 or the construction p*cadA* were introduced into WT and Δ*adiA*Δ*cadA*Δ*speF*. The resulting strains were compared during growth at pH 4.5 in the presence of 5 mM lysine (Figure 3). When growth was performed in anoxic conditions, the complementation by *cadA* improved the growth of Δ*adiA*Δ*cadA*Δ*speF* (see Δ*adiA*Δ*cadA*Δ*speF* p*cadA* versus Δ*adiA*Δ*cadA*Δ*speF* pACYC177) (Figure 3A). Growth of Δ*adiA*Δ*cadA*Δ*speF* p*cadA* was similar to WT p*cadA* but both strains displayed a lag compared to WT pACYC177. This suggested a potential toxicity associated with the expression of *cadA* from a low copy number plasmid in anoxic conditions. When growth was performed in aerobic conditions, the complementation by *cadA* restored the growth of Δ*adiA*Δ*cadA*Δ*speF* to the level of WT while Δ*adiA*Δ*cadA*Δ*speF* pACYC177 was still impaired (Figure 3B). Strains harboring p*cadA* did not show growth improvement at pH 4.5 if lysine was not provided into the medium (data not shown). To confirm that CadA alone was able to support growth without AdiA and SpeF, and to avoid toxicity associated with the expression of *cadA* from a low copy plasmid in anoxic conditions, we analyzed growth of the Δ*adiA*Δ*speF* strain at pH 4.5 in anoxic and aerobic conditions. In presence of lysine, the Δ*adiA*Δ*speF* strain was able to grow like the wild-type strain, showing the efficient contribution of CadA (Figure 3C and D). Next, survival of the Δ*adiA*Δ*cadA*Δ*speF* p*cadA* strain was examined at extreme acidic pH and compared to the control strains. When lysine was available during the challenge of one hour at pH 2.3, Δ*adiA*Δ*cadA*Δ*speF* p*cadA* showed survival while Δ*adiA*Δ*cadA*Δ*spe* pACYC177 did not (Table 3). The level of survival of Δ*adiA*Δ*cadA*Δ*spe* p*cadA* was comparable to WT, WT pACYC177 or WT p*cadA*. On the other hand, Δ*adiA*Δ*cadA*Δ*speF* p*cadA* had a poor survival compared to the other strains when arginine and ornithine were made available into the medium during the challenge. These results indicated that the survival observed in presence of p*cadA* was specifically dependent of the use of lysine.

**Table 3:** Survival after a stress of one hour at pH 2.3 when Δ*adiA*Δ*cadA*Δ*speF* is complemented by *cadA*.

Strain	Available substrate			
	None	Arginine	Lysine	Ornithine
**WT pACYC177**	0.6±0.4	74±25	33±9	10±0.7
**WT p** ***cadA***	2±2	86±42	32±11	24±6
**Δ** ***adiA*** **Δ** ***cadA*** **Δ** ***speF*** ** pACYC177**	0.07±0.1	<0.05	<0.05	0.08±0.08
**Δ** ***adiA*** **Δ** ***cadA*** **Δ** ***speF*** ** p** ***cadA***	2±1.7	1.8±0.2	35±11	2±1.8

Values in the table are percentage survival ± standard deviation, which results from three independent experiments. Viability was assessed by CFU count on LB plates. The percentage survival represents the ratio between the CFU counted after and before stress. Cultures and stresses were done in anoxic conditions. When indicated 20 mM L-Arg, L-Lys or L-Orn were added to the medium during the challenge.

Taken together, these experiments indicated that CadA alone was sufficient to carry out growth and survival at acidic pH as long as its cognate substrate was available.

### Growth improvement is accompanied by a deacidification of the environment

We wished to determine if, during growth at pH 4.5, the activity of decarboxylases influenced the environmental pH through the consumption of protons and the release of diamines. We focused on activities of lysine and ornithine decarboxylases as we just showed that the arginine decarboxylase did not confer any advantage in mild acidic conditions. The pH of the external medium was measured after 5 hours of growth. Cultures of the WT and Δ*adiA*Δ*cadA*Δ*speF* strains were started at pH 4.5 with or without addition of lysine and ornithine into the medium. After 5 hours of growth in anoxic conditions, the external pH value of the WT culture had slightly decreased to 4.3±0.05 when no substrates were made available in the medium, while it had reached 5.3±0.08 when lysine and ornithine were added at 5 mM final each. In contrast, the external pH value of the Δ*adiA*Δ*cadA*Δ*speF* culture solely decreased to 4.2±0.06 whether or not the substrates were present. The increase of the external pH was therefore related to the activity of the decarboxylases and not to the addition of the substrates. According to the Sørensen definition pH  =  -log_10_[H^+^], the activities of the lysine and ornithine decarboxylases led to 10 times less protons in the medium.

Taken together, these results suggested that, at moderate acidic pH, activities of lysine and ornithine decarboxylases allowed buffering of the environment and growth improvement.

### Construction of a reporter system to follow acidification of the external milieu

**Figure 4:**
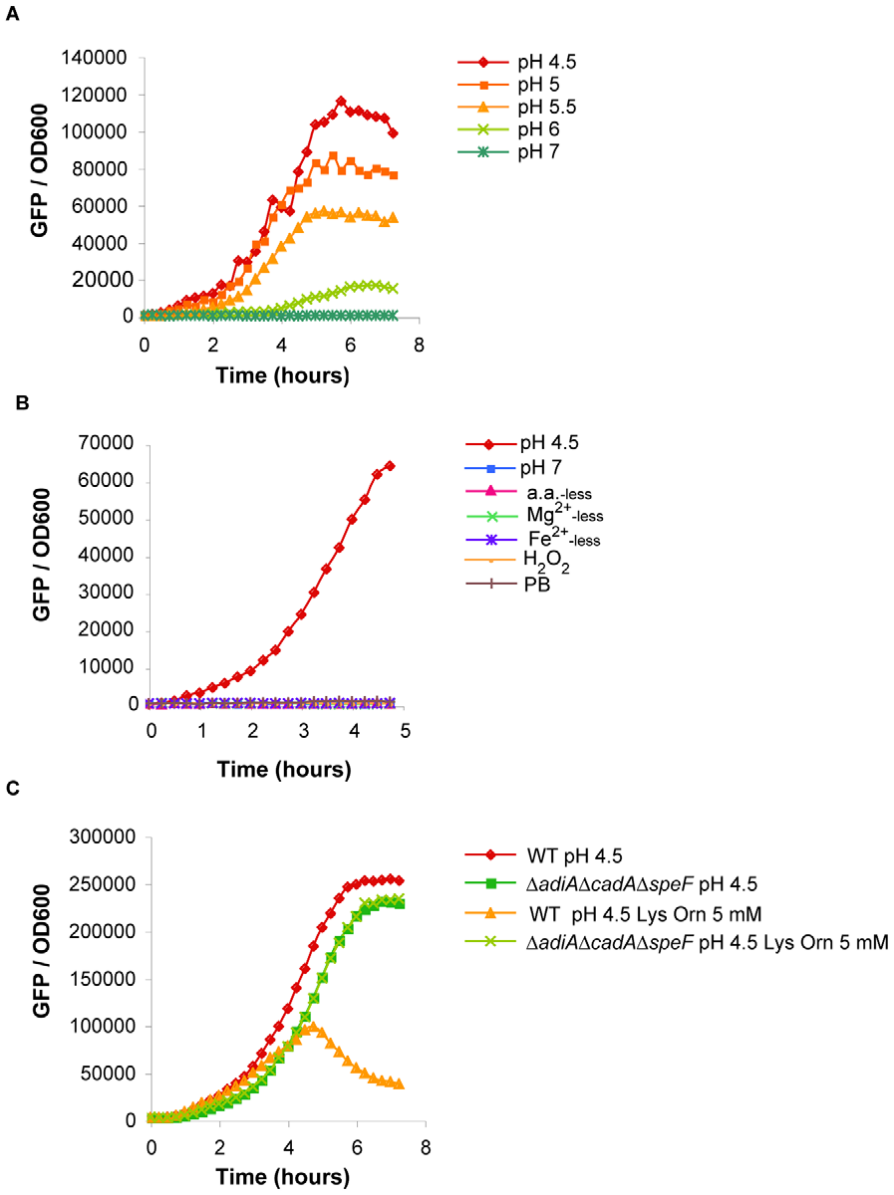
Characterization of the transcriptional fusion P*asr*::*gfp* in acidified M9 medium. Fluorescence produced by WT pP*asr*::*gfp* was monitored with a fluorometer and expressed proportionally to the bacterial population (GFP/OD_600_). **A**. WT pP*asr*::*gfp* was grown overnight in M9 medium complemented with 0.1% casamino acids, 0.2% glucose pH 7.2, diluted 1/50 and grown in the same medium adjusted to the desired pH with HCl. **B**. Bacteria were prepared as in A and grown in M9 medium adjusted to pH 4.5 or left at pH 7.2 with addition of different stresses: amino acids, magnesium or iron starvations (a.a._-less_, Mg^2+^_-less_ and Fe^2+^_-less_, respectively), oxidative stress (hydrogen peroxide : H_2_O_2_) and antimicrobial peptide (polymixin B : PB). **C**. WT pP*asr*::*gfp* and Δ*adiA*Δ*cadA*Δ*speF* pP*asr*::*gfp* were grown overnight in the same medium as in A, diluted 1/50 and grown in the same medium containing, when indicated, 5 mM L-lysine and 5 mM L-ornithine and adjusted to pH 4.5. Cultures were performed in anoxic conditions.

Biochemical reactions catalyzed by inducible amino acid decarboxylases eventually increased the external pH value, as we just showed in the paragraph above. We wished to investigate whether bacteria did perceive the change in the pH values of their immediate surrounding, mediated by the activity of the decarboxylases. For that purpose, we first made and characterized a reporter fusion designed to respond to external pH. The promoter of the *asr* gene was chosen based on studies indicating that (i) in *E. coli*, transcription of the *asr* gene was dependent upon external pH values within the range 5-4 [25], and was maximal at pH 4 [22], [26], and (ii) in *S.* Typhimurium, *asr* was the most induced gene into host macrophages as suggested by global transcriptional analysis [18]. The promoter of *asr* was fused to the gene of the Green Fluorescent Protein (GFP) on the pFPV25 plasmid. The construction, called pP*asr::gfp*, was introduced into *S.* Typhimurium and GFP fluorescence was followed as an indicator of environmental acidity. We first checked that the reporter fusion responded to acidic pH in the culture medium. WT pP*asr::gfp* was grown in M9 medium acidified within the pH range 7 to 4.5 with HCl and GFP fluorescence was measured with a fluorometer. As shown in Figure 4A, fluorescence was rapidly detected in bacteria grown at pH≤5.5 and fluorescence intensity increased with pH acidity going from 5.5 to 4.5. In control experiments, we checked that the reporter fusion was responding to none of the following treatments: addition of antimicrobial peptide, amino acids, Mg^2+^ or Fe^2+^ starvations and oxidative stress (Fig. 4B).

Last, we tested if the reporter fusion responded adequately to the deacidification of the surrounding environment. As mentioned in the paragraph above, activities of lysine and ornithine decarboxylases resulted in an increase of the environmental pH. WT pP*asr::gfp* was grown in M9 medium acidified to pH 4.5 in presence or not of lysine and ornithine. When lysine and ornithine were available in the medium, global fluorescence first increased, then, slowed down and finally dropped after 5 hours of growth at pH 4.5 (Fig. 4C). In contrast, when the same experiment was performed with Δ*adiA*Δ*cadA*Δ*speF* pP*asr::gfp*, the fluorescence signal increased throughout the time. The interpretation of these results was that the deacidification of the medium, by WT bacteria, finally turned off the *asr* promoter and stopped GFP production. Consequently, as the number of GFP molecules remained constant while the bacterial population was growing, the average fluorescence per bacteria decreased. When the Δ*adiA*Δ*cadA*Δ*speF* strain was cultured in presence of lysine and ornithine, deacidification could not occur, the *asr* promoter was continuously on and global fluorescence increased accordingly (Fig. 4C). Taken together, these results indicated that the activity of lysine and ornithine decarboxylases led to deacidification of the surrounding medium that could be perceived by the reporter strain.

### Activities of the decarboxylases influence the pH of the SCV

**Figure 5:**
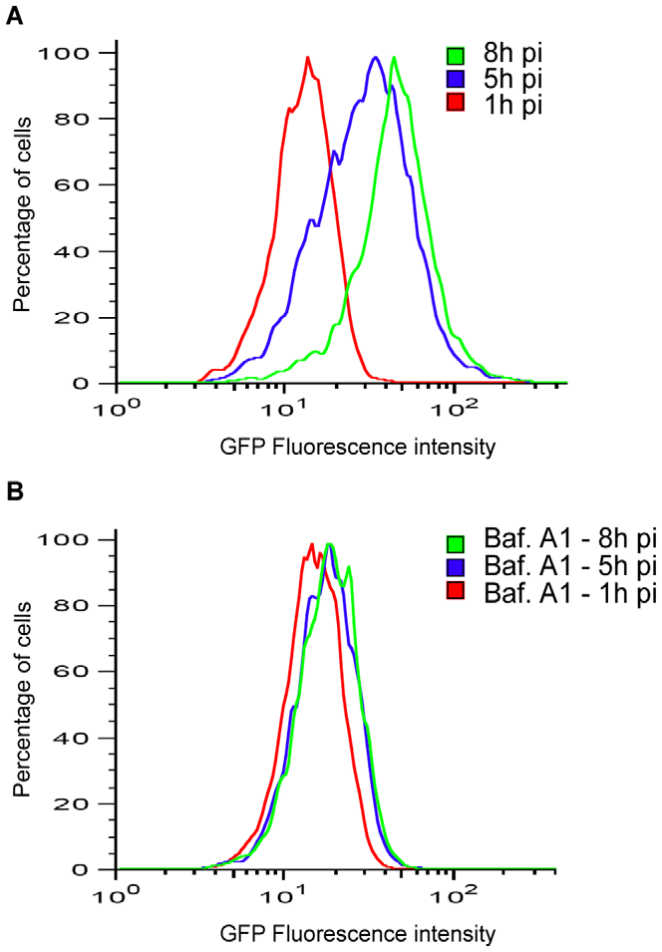
Response of the P*asr*::*gfp* transcriptional fusion during macrophage infection. WT pP*asr*::*gfp* was used to infect RAW264.7 macrophages. At various times post-infection (pi) host cells were lysed, bacteria were fixed with paraformaldehyde and collected. Fluorescence produced by intracellular WT pP*asr*::*gfp* 1, 5 and 8 hours post-infection was analyzed by flow cytometry (A). The same analysis was performed when host cells were treated with 100 nM bafilomycin A1, which inhibits SCV acidification (B). Typical graphs representative of several experiments are shown.

We wished to determine if the activity of decarboxylases would influence the pH of the SCV. For that purpose we used the capacity of *S.* Typhimurium to perceive its environment. In a first experiment, we examined if the SCV was perceived as an acidic environment. The reporter strain previously described, WT pP*asr::gfp,* was used to infect RAW264.7 macrophages. Host cells were lysed 1 hour, 5 hours and 8 hours post-infection and the fluorescence of intracellular bacteria was analyzed by flow cytometry. Fluorescence of intracellular bacteria increased during infection (Fig. 5A), indicating that the reporter strain perceived acidity of the SCV environment. As acidification of the SCV results from the activity of the vacuolar H^+^-ATPase, which delivers protons to the lumen of the SCV [5], we controlled the response of the reporter strain in presence of the vacuolar H^+^-ATPase inhibitor bafilomycin A1 [27], [28]. Fluorescence of WT pP*asr::gfp* did not increase during infection when host cells were treated with bafilomycin A1 (Fig. 5B).

**Figure 6:**
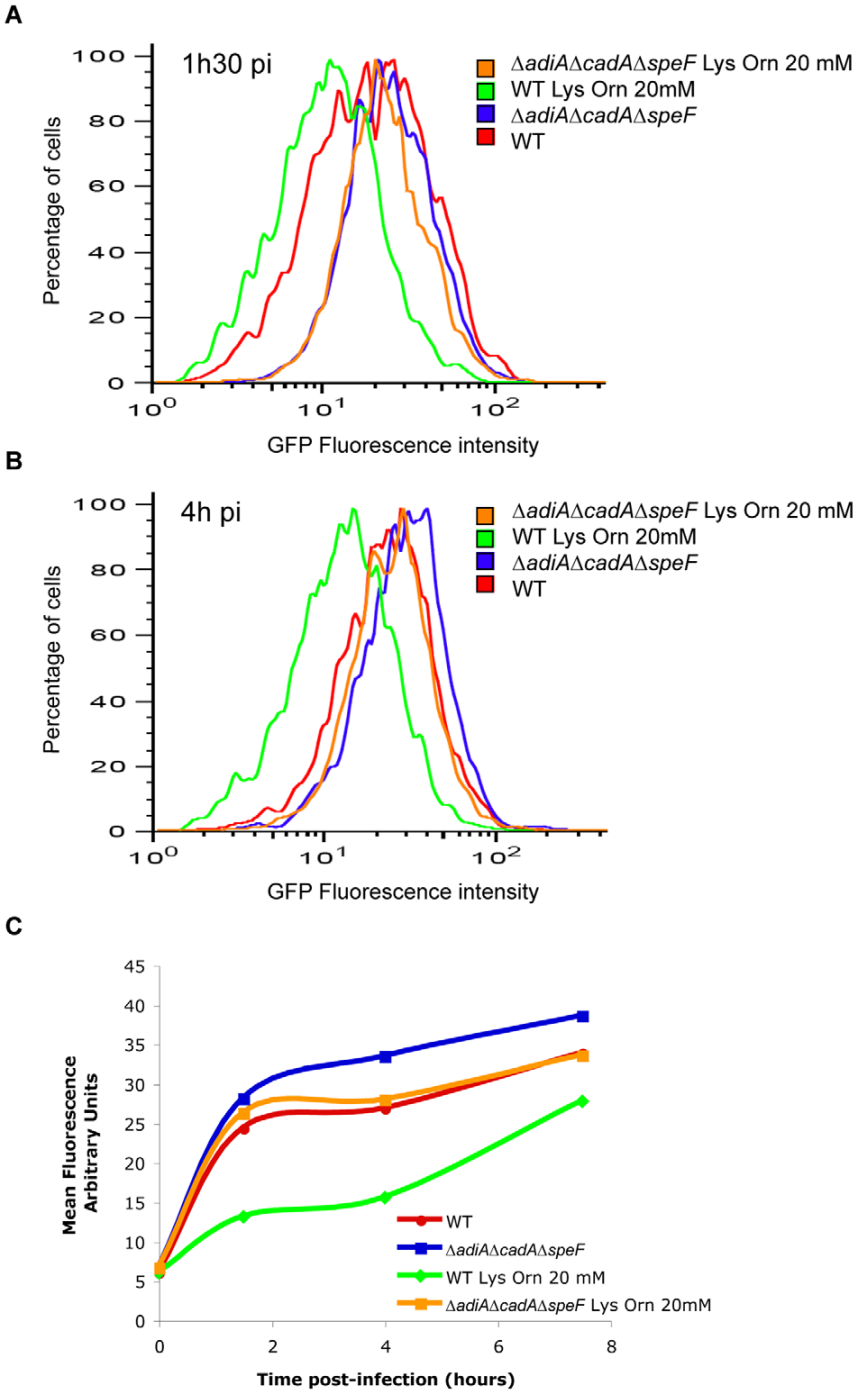
Delay in SCV acidification when decarboxylase activities are favoured. WT pP*asr*::*gfp* and Δ*adiA*Δ*cadA*Δ*speF* pP*asr*::*gfp* were grown for 5 hours in anoxia in LBG and used to infect RAW264.7 macrophages. When indicated L-lysine and L-ornithine, 20 mM each, were added in the cell culture medium 3 hours before infection and throughout the experiment. At 1h30 (A, C), 4h (B, C) and 7h30 (C) post-infection, macrophages were lysed, bacteria were fixed with paraformaldehyde and collected. Fluorescence produced by WT pP*asr*::*gfp* and Δ*adiA*Δ*cadA*Δ*speF* pP*asr*::*gfp* was analyzed by flow cytometry. In C, curves were produced using the mean FL1-H corresponding to the mean fluorescence intensity of the bacterial population for one time point.

Next, we determined if the activity of decarboxylases would modify the acidification of the SCV as perceived by the reporter strain. For that purpose we infected RAW264.7 macrophages with the WT pP*asr::gfp* and Δ*adiA*Δ*cadA*Δ*speF* pP*asr::gfp* strains. Intracellular bacteria were collected 1h30 and 4 hours post-infection and their fluorescence measured by flow cytometry. The fluorescence of Δ*adiA*Δ*cadA*Δ*speF* pP*asr::gfp* was always higher than WT pP*asr::gfp* (Fig. 6, blue and red lines, respectively), suggesting that the activity of the *asr* promoter had been turned on sooner as a result of earlier acidification of the SCV in the absence of the decarboxylases. When the amino acids ornithine and lysine were added in the cell culture medium, the fluorescence produced by WT pP*asr::gfp* was considerably lowered (Fig. 6, green line), indicating that the activity of the *asr* promoter turned on with a delay. This suggested that, when the substrates of decarboxylases were abundant, the SCV acidification was significantly delayed by the WT strain. Altogether, these results indicated that the activity of the decarboxylases influenced the pH of the SCV provided that their cognate substrates were available.

### Lack of the three inducible amino acid decarboxylases does not impair systemic infection

To determine if amino acid decarboxylases were important during infection we performed competitive index between WT and mutant strains. Bacteria were orally inoculated to use the route through the stomach. The mouse model of systemic infection, in which bacteria grow inside SCV into cells of the monocytic lineage, was used. Our goal was to examine if inducible amino acid decarboxylases allowed to survive the pH of the stomach and/or to favor growth into the acidified SCV.

**Figure 7:**
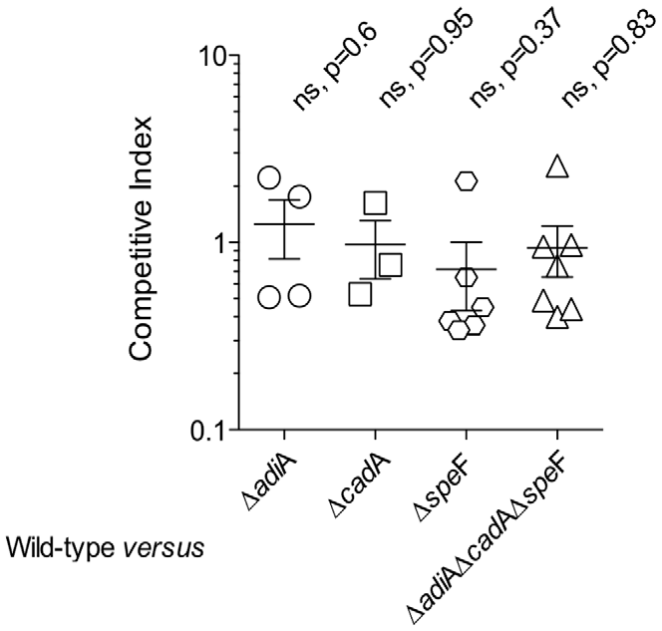
Competitive index in mice between WT and mutants of the inducible amino acid decarboxylases. Mice were orally inoculated with an equal mix of wild-type and mutant strains, spleens were analyzed 5 days post-inoculation. The CI was calculated as the output ratio of mutant to wild-type bacteria divided by the input ratio. Open symbols illustrate individual CI values, horizontal bars indicate means of CI values and error bars represent the standard error of the mean. Abbreviation ns means not significantly different from wild-type, which occurs when p-value (p) is superior to 0.05. The Δ*adiA*Δ*cadA*Δ*speF* strain is the kanamycin resistant version of Δ*adiA*Δ*cadA*Δ*speF* (strain n° 199).

Fasted mice were inoculated with a mix containing an equal amount of WT and one of the four mutants Δ*adiA*, Δ*cadA*, Δ*speF* or Δ*adiA*Δ*cadA*Δ*speF*. To make substrate(s) available in the stomach, 20 mM of amino acid substrate(s), susceptible to be used by the WT but not by the mutant strain, were delivered with the bacterial inoculum. The bacterial populations of the spleens were analyzed 5 days post inoculation. Our results indicated that the ratio of mutant to wild-type varied between twice more and twice less in most of the individual CIs with p-value superior to 0.05 for each set of competitive index (Figure 7). This led us to conclude that none of the mutants was significantly out-competed by the WT strain. These results indicated that, in the mouse model of systemic infection, decarboxylases seemed to be dispensable for virulence.

## Discussion

Three inducible amino acid decarboxylases are found in *S*. Typhimurium, AdiA, CadA and SpeF, each using arginine, lysine and ornithine as a substrate, respectively. In this study, the contribution of the three enzymes, in *S*. Typhimurium's adaptation to extreme and moderate acidic pH, was compared in the exact same conditions for the first time. Our results indicated that all three inducible amino acid decarboxylases improved survival at pH 2.3, although to different extents, and two of them also improved growth at pH 4.5. Evidences were obtained that confirm their capacities to buffer pH of bacterial surrounding. Nevertheless, no evidences were obtained for these enzymes to have a prominent role during systemic infection.

A *S*. Typhimurium Δ*adiA*Δ*cadA*Δ*speF* strain devoid of all three inducible amino acid decarboxylases was severely impaired in acid resistance at pH 2.3 and unable to improve growth at pH 4.5, definitively establishing a role for these enzymes in allowing *S*. Typhimurium to keep up with acid stress. However, contributions of each of the three decarboxylases differed: (i) the arginine decarboxylase, AdiA, played a predominant role for survival at extreme acidic pH (pH 2.3) but was helpless during growth at moderate acidic pH (pH 4.5); (ii) the ornithine decarboxylase, SpeF, improved growth significantly at moderate acidic pH in absence of oxygen only, but played a minor role during survival; and (iii) the lysine decarboxylase, CadA, had a broader range of actions and conferred both significant survival at pH 2.3, and growth improvement at pH 4.5 in an O_2_-independent manner. Several parameters would have to be investigated to decipher the molecular basis of these differences. One is regulation of their synthesis and on this aspect attention should be brought to oxygen conditions. Indeed, we, and others, noticed that resistance at very acidic pH conferred by inducible decarboxylases was conditioned by oxygen limitation during culture and stress [13] (our data not shown). In addition, our results indicated that the growth advantage conferred by the inducible ornithine decarboxylase at mild acidic pH was only observed in anoxic conditions. This is consistent with the observation that anoxic conditions promote expression of the genes encoding the inducible amino acid decarboxylases [13], [24]. A careful analysis of the impact of oxygen, on the activity of each of the three promoters, would have to be performed along with a study of the mechanism of regulation. Another parameter to explore is regulation of their activity as they do exhibit differences in their optimum pH value. The activity of SpeF (optimum pH of 7 [29]), would be favored when the internal pH is near neutrality while the activity of AdiA (optimum pH of 5.2 [30]), would be favored when the internal pH drops to 5. In agreement with that, the bacterial cytoplasm is indeed maintained in a physiological range over an external pH range of 5-7 but it drops to pH 4-5 when external pH reaches extreme values such as pH 2-3 [14], [31], [32].

Over the panel of acid stress conditions, the Δ*adiA*Δ*cadA*Δ*speF* strain was the most altered of the mutant strains. Interestingly, we noticed that Δ*adiA*Δ*cadA*Δ*spe* had a worst survival at pH 2.3 than WT even in the absence of extracellular amino acids. We therefore suggest that inducible amino acid decarboxylases could use the internal pool of amino acids when bacteria are challenged to death. The level of protection would however be restricted by the amount of internal amino acids available, or, the level of induction of decarboxylases, which can be dependent on substrate concentration [14], [15].

Activities of inducible amino acid decarboxylases coupled to activities of their corresponding antiporters allow consumption of protons and amino acids and release of amines in the external medium. Their activity was therefore expected to buffer the immediate bacterial surrounding, which we showed by measure of the pH of the external medium. The next question was to determine if these enzymes could influence the pH of the SCV.

A great advantage linked to the use of *S*. Typhimurium as a model of intracellular pathogens is the availability of cellular and animal models. However, characteristics of the environment wherein an intracellular bacterial pathogen develops can be quite difficult to describe. We chose to explore the SCV environment as perceived by *S.* Typhimurium. We developed and characterized a reporter transcriptional fusion designed to respond to acidification of the environment. Though by this approach we followed the activation of only one promoter, we avoided problems usually linked to the use of chemical probes such as efficiency of membrane permeability, precise cellular localization, signal-to-background ratio and necessity of time-lapse video. Characterization of the strain, carrying the P*asr*::*gfp* transcriptional fusion, in culture media, showed that fluorescence was produced specifically in response to acidity and to none of a series of other stresses susceptible to be met by the bacterium inside its host, such as amino acids, Mg^2+^ or Fe^2+^ starvations, oxidative stress or presence of antimicrobial peptides. Inside macrophages, the fluorescence of the reporter strain increased substantially. This response was abolished by an inhibitor of vacuolar acidification, thereby establishing that *S.* Typhimurium perceives the SCV as an acidic environment. Inside macrophages, WT pP*asr*::*gfp* produced less fluorescence compared to Δ*adiA*Δ*cadA*Δ*speF* pP*asr*::*gfp* indicating that acidification of the SCV arose sooner in the absence of inducible amino acid decarboxylases.

Next, we wished to examine if inducible amino acid decarboxylases could confer an advantage to *S*. Typhimurium during infection. As we showed that acidification of the SCV could be delayed by the activity of the inducible amino acid decarboxylases, we first attempted to assess if the proliferation of Δ*adiA*Δ*cadA*Δ*speF* was impaired inside macrophages. Our assays indicated that intracellular proliferation of WT and Δ*adiA*Δ*cadA*Δ*speF* was about the same (data not shown). Reasoning that the amino acid content of the SCV might not be sufficient to reveal the potential role of inducible amino acid decarboxylases, we added 20 mM lysine and ornithine into the medium. However, in a proliferation assay time-scale, addition of those amino acids led the cells to lift off and results could not be interpreted. We therefore chose to examine if inducible amino acid decarboxylases contributed to virulence using the mouse model of systemic infection. The systemic model of infection by oral route was chosen as it brings together the passage through the acidic stomach of the infected animal and proliferation inside mildly acidified SCV into cells of the monocytic lineage. The acidity of the stomach is cited as the first line of protection against swallowed microbes and infectious dose of a food-borne bacterial pathogen is often correlated to its capacity to resist extreme acidic pH [1], [12]. In the context of our clear establishment of a role for decarboxylases in protecting *S.* Typhimurium exposed to acid stress when challenged in synthetic media, it was unexpected to find that the presence of decarboxylases did not confer any advantage for virulence. A first possibility to account for this discrepancy might be related to our observation that decarboxylases provide an advantage in quite a narrow pH range. Indeed, whereas the survival of WT was considerably higher than Δ*adiA*Δ*cadA*Δ*speF* at pH 2.3, both strains were similarly killed at pH 1.5 or resistant at pH 3 (data not shown). Thus, while it is easy to precisely control the pH of synthetic media, the pH value of the stomach is much more subject to uncertainties due to intrinsic variations, since the pH value of the stomach is likely to fluctuate between 1 and 5 at the beginning of digestion, or due to extrinsic factors such as the delivery of the inoculum. A second possibility is related to oxygen content. Since oxygen content decreases through the intestine, it could be high enough in the stomach to not promote an optimal expression of the decarboxylases. Independently of the underlying molecular mechanisms, our results do not show a role for the arginine, lysine and ornithine decarboxylases in the systemic mode of infection. These results do not preclude a possible role for these enzymes in other modes of infection such as gastroenteritis or persistence that we did not test. Actually, the conclusion that the lack of inducible amino acid decarboxylases did not affect systemic infection might meet with consideration about possible ongoing genome reduction in relation with the mode of infection. Indeed, as inducible amino acid decarboxylases seemed to have no role during systemic infection, we examined the genomes of *Salmonella* species responsible of systemic infection such as *S*. Typhi and *S*. Paratyphi and observed that those enzymes were poorly conserved. The analysis of the genomes of 16 typhoid and non-typhoid *Salmonella* species available on the Kyoto Encyclopedia of Genes and Genomes (http://www.genome.jp/kegg/) showed that the gene encoding the ornithine decarboxylase evolved as a pseudogene in *S.* Typhi, and so did the gene encoding the arginine decarboxylase in *S.* Paratyphi C and the gene encoding CadC the positive regulator of the lysine-dependent decarboxylase/antiporter system, in *S.* Paratyphi A. Moreover, no role in virulence could be attributed to these three inducible amino acid decarboxylases in other bacteria [33], [34]. Even more, in *Shigella,* inactivation of the lysine-dependent system is a trait of the *Shigella*/EIEC pathotype. Such an inactivation has been proposed to correspond to a pathoadaptative mutation, regarded as a step in obtaining full invasiveness potential [35], [36].
